# P-631. A Practice-Based and Expert-Driven Consensus on the Management of Respiratory and Non-Respiratory Adverse Events Associated with Amikacin Liposome Inhalation Suspension (ALIS)

**DOI:** 10.1093/ofid/ofaf695.844

**Published:** 2026-01-11

**Authors:** Colin Swenson, Charles L Daley, Jennifer Faber-Gerling, Patrick A Flume, Shannon H Kasperbauer, H Jeffrey Kim, Amy Leitman, Theodore K Marras, Kozo Morimoto, Kenneth N Olivier, Kevin L Winthrop, Juzar Ali

**Affiliations:** Emory University, Atlanta, GA; National Jewish Health, Denver , CO; National Jewish Health, Denver, CO, denver, Colorado; Medical University of South Carolina, Charleston, SC, USA, charleston, South Carolina; National Jewish Health, Denver, CO, denver, Colorado; Georgetown University Hospital, Washington, DC, USA, washington, Washington; NTM Info & Research, Palmetto Bay, FL, USA, Palmetto Bay, Florida; University Health Network and University of Toronto, Toronto, Ontario, Canada; Fukujuji Hospital, Tokyo, Tokyo, Japan; University of North Carolina School of Medicine, Chapel Hill, NC, USA, Chapel Hill, North Carolina; OHSU-PSU School of Public Health, Portland, Oregon; Louisiana State University Health Sciences Center, New Orleans, LA, USA, New Orleans, LA

## Abstract

**Background:**

Amikacin liposome inhalation suspension (ALIS), a nebulized formulation of amikacin, is the first treatment approved in the United States, as part of a combination therapy, for adults with refractory *Mycobacterium avium* complex lung disease (MAC-LD) who have limited or no alternative treatment options. In clinical studies, ALIS treatment was associated with adverse events (AEs) of mild or moderate severity which led to treatment discontinuation in up to 24.4% of patients. In clinical practice, inadequate management of AEs may lead to decreased treatment adherence and early discontinuation.

An expert steering committee (SC) convened to establish practice-based, and expert-agreed recommendations for the mitigation strategies of ALIS-related AEs for patients with MAC-LD, with the aim of providing up-to-date practical recommendations for healthcare professionals.

**Methods:**

The SC included: 7 pulmonologists, 2 infectious disease specialists, 1 otolaryngologist, 1 nurse practitioner, and 1 patient advocate. The SC aligned on clinical topics and identified critical unanswered questions. Recommendations were drafted to address each question based on a comprehensive literature review and clinical experience with patients. Consensus on recommendations for each statement was achieved via modified Delphi methodology voting on a 9-point scale.

**Results:**

The SC identified the following clinical topics: preparing the patient before the start of treatment; preventing AEs; monitoring AEs; mitigating AEs and incorporating the assistance of a multidisciplinary team. Due to the paucity of publications, most recommendations are expert-opinion based.

**Conclusion:**

The SC made recommendations based on literature, healthcare experience, and patient perspectives for the management of ALIS-associated AEs. A standardized team approach and involvement of a multidisciplinary team, including pulmonologists, infectious disease specialists and otolaryngologists, may help facilitate adherence to treatment. It is expected that successful recognition and management of AEs will be aided by educating patients and caregivers, and informed members of the clinical care team before and during ALIS treatment.

This initiative was funded by Insmed Inc. with no input on the recommendations.

**Disclosures:**

Colin Swenson, MD, Insmed Inc.: Advisor/Consultant Charles L. Daley, MD, AN2: Advisor/Consultant|AN2: Grant/Research Support|Bill and Melinda Gates Foundation: Advisor/Consultant|Bugworks: Grant/Research Support|Galapagos: Advisor/Consultant|Grifols: Advisor/Consultant|Hyfe: Advisor/Consultant|Insmed Inc.: Advisor/Consultant|Insmed Inc.: Grant/Research Support|Juvabis: Grant/Research Support|MannKind: Advisor/Consultant|MannKind: Grant/Research Support|Matinas BioPharma Holdings, Inc.: Advisor/Consultant|Monaghan: Advisor/Consultant|Nob Hill: Advisor/Consultant|Ostuka Pharmaceutical: Advisor/Consultant|Paratek Pharmaceuticals: Advisor/Consultant|Paratek Pharmaceuticals: Grant/Research Support|Shionogi: Advisor/Consultant|Spero Therapeutics: Advisor/Consultant|Spero Therapeutics: Grant/Research Support Jennifer Faber-Gerling, NP, Insmed Inc.: Advisor/Consultant Patrick A. Flume, MD, Insmed Inc.: Advisor/Consultant|Insmed Inc.: Grant/Research Support Shannon H. Kasperbauer, MD, AN2: Advisor/Consultant|COPD Foundation: Advisor/Consultant|Insmed Inc.: Advisor/Consultant|Paratek: Advisor/Consultant|Zambon: Advisor/Consultant H. Jeffrey Kim, MD, Insmed Inc.: Advisor/Consultant Amy Leitman, N/A, MicuRx: Advisor/Consultant Theodore K. Marras, MD, Insmed Inc.: Advisor/Consultant|Mannkind Corp: Advisor/Consultant|Partner Therapeutics: Advisor/Consultant Kenneth N. Olivier, MD, Insmed. Inc: Advisor/Consultant|Mannkind Corporation: Advisor/Consultant|Paratek Pharma: Advisor/Consultant|Paratek Pharma: Site PI|ReCode Therapeutics: Grant/Research Support|Spero Therapeutics: Advisor/Consultant|Spero Therapeutics: Site PI|Verona Pharma: Site PI Kevin L. Winthrop, MD, MPH, Insmed Inc.: Grant/Research Support Juzar Ali, MD; FRCP(C); FCCP , EuroImmun: Honoraria|Insmed Inc.: Advisor/Consultant

